# Supplementary material for: Leveraging a synthetic biology approach to enhance BCG-mediated expansion of Vγ9Vδ2 T cells
Source: PLoS One. 2026 Apr 6;21(4):e0343925. doi: 10.1371/journal.pone.0343925 (PMC13052839; doi:10.1371/journal.pone.0343925)
Supplement: Attachment — Submitted filename: Response to reviewer comments.docx [file pone.0343925.s006.docx]

**Point-by-point response to reviewers**

We are thankful for the reviewers’ time and attention in reading our manuscript carefully, and for the very helpful suggestions and ideas. We have addressed all of the points raised, mostly by incorporating new experimentation to address the most major comments. In particular we have performed four new experiments that include new metabolomics data and that resulted in the inclusion of a new figure (Fig 4). Below is our point-by-point response to each of the reviewers’ concerns.

**Reviewer #1:** In this paper, Qabar CM et. al. report the generation of a recombinant M. bovis BCG strain that overexpresses an isoprenoid metabolite and phospho-antigen known as HMBPP. HMBPP is a stimulus which expands Vγ9Vδ2 T-cell subsets in the host, and these same cells are believed to play an important role in containing TB disease. The authors postulate that an rBCG strain of this nature would be a more potent TB vaccine than standard BCG.

This concept is innovative and novel. This paper reports a preliminary, partial dataset to support their hypothesis.

Concerns

1. Fig. 3c. There is insufficient evidence to claim that overexpression of GcpE induces Vg9Vd2 cell expansion. The claim is based on Fig. 3c where we are shown on 2 determinations (BCG-WT) compared to 3 determinations (BCG+gcpE-OE). This sample size is too low. In contrast, Fig. 2c (5 dilutions tested on multiple replicates) is convincing evidence that BCG+MEPsynth strain does not elicit the intended Vγ9Vδ2 response. However, we are not shown the same 5 dilutions tested on multiple replicates for the BCG+gcpE-OE strain. The authors should either show more data for the GcpE strain or withdraw the claim that the GcpE overexpressor induces Vg9Vd2 cell expansion. *To address this concern, we performed more expansion assays with two new PBMC donors and the results of these experiments are consistent with previous data and support our previous conclusions.* ***This new data is incorporated into Figs 2 and 3, as well as the supplement.***
2. Fig 3c. Why are we only shown the results from Donor E? The legend indicates there was a second donor tested. *For clarity, we show data from one donor in the main figures because of extensive donor to donor variability, which is common in clinical samples. All other donors are shown in the supplement, including two more experiments with new donors.*
3. Since the synthetic biology approach did not increase the expansion of Vγ9Vδ2 T-cells, the authors should consider modifying the title of the manuscript. *We now appreciate that there was a fair amount of donor-to-donor variability in our initial donor samples, and we included donors who were unresponsive even to purified HMBPP, our positive control. We have now included data only from donor PBMCs who are responsive to HMBPP, and have measured significant increases in Vγ9Vδ2 T cell expansion over baseline using the synthetic biology approach, which we have supported with further experimentation and have clarified in the text.*
4. In addition to measuring Vg9Vd2 cell expansion for the recombinant BCGs, it would be valuable to know what the actual levels of HMBPP are. *We attempted to measure HMBPP using LC-MS, however it was below our limit of detection in all samples. HMBPP has historically been difficult to measure (both in the literature and historically among our own lab and our collaborators), potentially due to the instability or high turnover rate of HMBPP.* ***We did, however, measure other MEP intermediates and critical cellular metabolites (NADH, NADPH, pyruvate, acetyl CoA, ATP) and have added these data as figure 4.***
5. Why were only BCG lysates tested in the Vγ9Vδ2 T-cell expansion assay and not the live BCG strains? *The expansion assay using lysates is well-established in the Vg9Vd2 literature (PMID 10886403, 15013758, 34381163, 12117907). We have not yet been able to establish a live infection model using mycobacteria that reliably induces Vg9Vd2 expansion.*
6. Figure 1 has several inconsistencies which should at the very least be discussed as limitations: (i) Why do the mutants show a growth defect in the absence of ATc-mediated knockdown. *While expression of the dCas9 enzyme is repressed under uninduced conditions, there is nonetheless some leaky expression that may result in low background levels of dCas9, and even low levels of essential gene repression is toxic.* (ii) Why does the “No guide control” exhibit a growth defect in presence of ATc. *ATc does have mild toxicity and slightly lengthens the growth rate of BCG.* (iii) While the spotting assay is a quick and dirty approach, to claim that a gene is essential, a CFU-based growth kinetic analysis should be done. *We have quantified CFU +/- induction, which clearly demonstrates that in the presence of inducer, targeting of these essential genes is lethal.* ***New data has been added to figure 1B****.* (iv) Lastly, qRT-PCR validation is necessary to show that the knockdowns are functioning correctly. *We assume that this comment is raising the possibility that the guides for both dxr and ispH are leading to cell toxicity due to off-target effects. We agree that this is a possibility, however, the likelihood of two guides both having off-target effects that inhibit growth seems extremely low. The qPCR experiment suggested by the reviewer would lend evidence that the CRISPRi guide is having a direct effect on dxr, but there could still be off-target effects that are not predictable. There is already overwhelming evidence that these genes are essential in other mycobacteria (PMID 18489786, 26309039, and 41165411) and this is not a major point of the paper.*
7. Fig 2b and 3b. Why are mRNA levels so low with these strong promoter constructs at 0.00015 (Fig. 2b) or 0.002 (Fig. 3b) relative to 16S rRNA? *Using 16s to normalize mRNA is standard in the field, and there are much higher levels of 16s rRNA in the cell compared to these targets, even with strong promoter elements. We have re-analyzed these data using a log2 fold change method, per the suggestion of Reviewer 2, which should clarify this concern as well.*
8. Fig. 2c and 2c should have statistics included. *Data were re-analyzed and statistics included.*
9. Inclusion of the flow cytometry dot plots and gating strategy for Fig. 2c and 3c would be helpful. ***These have been added to supplementary figures 1 and 2.***
10. Line 58-59: The statement that Vγ9+Vδ2+ γδ T cells increase to ~50% of total blood T cells should be qualified. Other studies show lower values closer to 20% (eg Kroca et al. PMID: 10792377). *We have changed the language around this statistic to reflect that 50% is the upper range of what has been reported.*
11. Lines 207-210. Why do the authors think that dxr and ispH appear to be essential in BCG by CRISPRi in their hands, but that in Mtb transposon mutants were viable as reported by the DG Russell lab (ref 63). *Upon in depth analysis of their dataset, it appears that their transposon insertion sites were in lytB1 and dxs2, which were later determined to be non-essential gene duplicates by the Parish lab (PMID: 26309039, PMID: 20172995). We have changed the text to instead cite three other TnSeq datasets which identified MEP genes as essential.*
12. Line 220. The text states the essential gene rpoB was targeted but Fig 1b shows folC was targeted. Please clarify. *We have corrected this in the text; we targeted rpoB in a separate project.*
13. Line 329. Discussion should be toned down. Feedback inhibition was a possible reason, but this was not formally demonstrated. *We have edited the discussion accordingly.*
14. The approach for increasing HMBPP used herein was essentially “make more” or “turn up the faucet”. In the discussion, it might be worth mentioning that another way to increase HMBPP levels in BCG would be to conditionally knockdown ispH and thereby reduce usage or “close the drain”. *We have updated the discussion to include this great suggestion!*

**Reviewer #2:** The manuscript presents a novel synthetic biology-based strategy to enhance TB vaccine efficacy by engineering the MEP pathway in BCG to increase HMBPP production, thereby stimulating Vγ9Vδ2 T cells. This concept is timely and innovative, combining metabolic engineering with immune modulation to address current limitations in TB vaccination strategies. The rationale and design are well-justified, and the use of human PBMCs enhances translational relevance. However, several critical revisions are required to ensure methodological rigor, data transparency, and appropriate interpretation:

A. Methodological Transparency: Clearly indicate sources of strains, plasmids, antibodies, and include gene identifiers such as Rv numbers or BCG locus tags. *We have added BCG locus tags to the methods section and the other requested information is located in the corresponding methods section and in supplementary tables.*
B. CRISPRi Knockdown Assay – Figure 1B
   a. The spot assay lacks proper controls and normalization:
      i. Include a dCas9-only or scrambled sgRNA control. *What we labeled “No guide control” is indeed the dCas9-only control. We have updated the figure for clarity.*
      ii. Ensure equal plating, incubation timing, and inoculum across plates. The no-guide control shows reduced growth upon ATc induction, indicating possible non-specific effects of dCas9 or ATc, which compromises interpretation of gene-specific knockdowns. *We have quantified CFU +/- induction, which clearly demonstrates that in the presence of inducer, targeting of these essential genes is lethal.* ***New data has been added to figure 1B****.* Additionally, unequal bacterial input and apparent differences in incubation timing between plates further confound comparison. *Bacterial input and incubation timing are uniform across plates and strains. We have updated the methods section to make this clear.* The absence of quantitative measurements (e.g., CFU counts or spot intensity) and replicates limits the robustness of the conclusions. *We have measured CFU; please see the next comment.*
      iii. Provide CFU counts or quantification of spot intensity across replicates. Without these controls, gene essentiality interpretations are weakened. *We have quantified CFU +/- induction, which clearly demonstrates that in the presence of inducer, targeting of these essential genes is lethal.* ***New data has been added to figure 1B.***
C. Gene Expression Analysis – Figures 2B and 3B
   a. The RT-qPCR data should be reanalyzed and presented using the ΔΔCt method.
      i. Display data as log₂ fold changes with appropriate statistical tests and annotations. This will improve clarity, comparability, and rigor. *We have updated the manuscript text, figures, and figure legend accordingly.*
D. PBMC Stimulation – Figures 2C and 2D
   a. Normalization of Lysates: OD₆₀₀-based normalization is insufficient:
      i. Include growth curves or CFU counts to demonstrate equal biomass. *We have included CFU data and pellet weights to demonstrate equal biomass and OD:CFU ratio, demonstrating the robustness of OD normalization.* ***New data has been added to supplementary figure 3.***
      ii. Specify how lysate input was standardized. *We have updated the methods section to specify the standardization.*
      iii. Authors are requested to quantify HMBPP levels via LC-MS or other methods to support biological interpretation. *We performed these experiments and unfortunately were not able to detect this metabolite. However, we note that HMBPP has been particularly difficult to quantify via LC-MS by others as well (e.g. PMID: 27480689), yet we are able to measure it by bioassay and have included further replicates using this assay. Additionally, we were able to measure other intermediates of this pathway via LC-MS, showing that the genetic perturbations don’t affect global cellular metabolism.* ***These new metabolomic data have been added as Figure 4.***
   b. Statistical Annotation (Figure 2D):
      i. Please include statistical comparisons between: § HMBPP vs. BCG WT § HMBPP vs. BCG+MEP^synth. Add p-values and update the legend accordingly. *We have updated the figure and appropriate legend accordingly.*
      ii. Clarify the type and number of replicates used (biological vs. technical) for each experiment.
          1. In particular, for the PBMC-based assays, the number of data points per group appears inconsistent across conditions—for example, in Figure 2D. If the assays were performed using PBMCs derived from the same set of human donors and stimulated simultaneously with different conditions, the number of samples (i.e., data points or “dots” per group) should be identical across all treatment groups. Please clarify the experimental setup and provide an explanation for these discrepancies in sample numbers. *Figures have been updated to show multiple dilutions per lysate; figure legends have been updated to clarify number of donors, replicate wells, and variability.*
E. Biosafety and Ethical Approvals
   a. The manuscript must clearly state Institutional Biosafety and IRB approvals for: Genetic manipulation. Use of human PBMCs. Include protocol numbers or justification for exemption, if applicable. *We have updated the methods section accordingly. Briefly, deidentified samples obtained from the Stanford Blood Center do not fall under use of human research subjects according to University of California, Berkeley Human Research Protection Program’s policy on Research with Coded Private Information or Biological Specimens. Further information can be found at* [*https://cphs.berkeley.edu/review.html#coded*](https://cphs.berkeley.edu/review.html#coded)*. Further, we have Institutional Biosafety approval for all biological agents and genetic manipulations under BUA #407 at UC Berkeley.*
F. Statistical Analysis
   a. Indicate: Statistical tests used in each figure, Exact p-values or significance thresholds. Confidence intervals where applicable. *We have updated figures and legends accordingly.*
G. Discussion
   a. Expand the discussion on potential limitations, possibility of feedback regulation in MEP pathway, how isoprenoid over-production alters the production of classical antigens of BCG. *We have updated the discussion accordingly.*
   b. Discuss absence of protective efficacy data. Reframe claims of protective immunity as speculative, pending validation in appropriate in vivo models. *We have updated the discussion accordingly.*
   c. If you are adding HMBPP quantification, please discuss that. *We have updated the discussion accordingly.*
   d. Strengthen context with references to:
      i. Clinical efforts involving Vγ9Vδ2 T cell-targeted vaccines. *We have updated the discussion accordingly.*
      ii. Metabolic engineering strategies in mycobacteria. *We have also updated the discussion to strengthen this point.*
H. Compliance with Journal Requirements
   a. Include detailed author contribution statements as per PLOS ONE guidelines. *Author contributions were completed in the PLoS One submission portal.*

Overall, the manuscript holds promise but requires substantial revision. Addressing these issues will significantly enhance the robustness and impact of the study.

**Reviewer #3:** The manuscript presents an innovative synthetic biology approach to enhance BCG-mediated expansion of Vγ9Vδ2 T cells by engineering the isoprenoid biosynthesis pathway in BCG, specifically to overproduce HMBPP. The underlying hypothesis is sound and the objective highly relevant in the context of TB vaccine enhancement strategies, particularly considering the unique role of Vγ9Vδ2 T cells in primate immunity. However, in its current form, the study appears somewhat inconclusive and requires additional data to substantiate its conclusions.

Major Concerns:

1) A critical shortcoming of the study is the lack of direct quantification of HMBPP in the engineered BCG strains. Although gene expression levels of MEP pathway genes and gcpE are provided, they cannot serve as a surrogate for actual metabolite levels. Without intracellular or extracellular quantification of HMBPP, the interpretation that gcpE overexpression leads to increased HMBPP accumulation remains speculative. It is imperative that the authors include HMBPP quantification (e.g., via LC-MS/MS or other validated methods) in both synthetic operon-expressing and gcpE-overexpressing strains to substantiate their claims. *We performed these experiments and unfortunately were not able to detect this metabolite. However, we note that HMBPP has been particularly difficult to quantify via LC-MS by others as well (e.g. PMID: 27480689), yet we are able to measure it by bioassay and have included further replicates using this assay. Additionally, we were able to measure other intermediates of this pathway via LC-MS, showing that the genetic perturbations don’t affect global cellular metabolism.* ***These new metabolomic data have been added as Figure 4.***

2) In Figures 2C, 2D, and 3C, the number of biological replicates (i.e., PBMC donors) and technical replicates is either unclear or inconsistently described in the legend. This limits the interpretability and statistical robustness of the expansion assay data. Authors should clearly state the number of independent donors, replicate wells, and how variability was accounted for in the analyses (mean ± SD, statistical tests, etc.). A separate sub-section on ‘Statistical Analysis’ should be added to ‘Materials and Methods’ section. *We have performed two more experiments from additional donors and have updated the main figures and supplement. Figures have been updated to show multiple dilutions per lysate; figure legends have been updated to clarify number of donors, replicate wells, and variability.* ***The methods section has been updated with a statistical analysis subsection.***

3) While expansion of Vγ9Vδ2 T cells is demonstrated, no functional immune response (e.g., cytokine release, or cytotoxic activity) is assessed to confirm that these expanded cells are immunologically competent. Inclusion of such functional data (IFNγ, Granzyme B, etc.) would strengthen the manuscript and its relevance to vaccine efficacy. *Other work has demonstrated that Vγ9Vδ2 effector functions were retained in ex vivo expansion assays from human PBMCs (PMID 38221530; PMID 34149689; PMID 29402645). Given that ex vivo HMBPP-expanded, human PBMC-derived Vγ9Vδ2 T cells have been shown to perform effector functions, and that our main goal is to demonstrate that engineering these strains specifically impacts expansion, we believe that functional immune profiling is beyond the scope of this study.* ***However, we have updated the discussion to reframe claims of protective immunity as speculative, pending in vivo validation.***

Minor Comments:

1) Reference [29] appears to have a non-standard font or formatting. This should be corrected. *We have corrected this.*
2) The manuscript frequently repeats the background on Vγ9Vδ2 T cells and HMBPP throughout the introduction and results. This dilutes the emphasis on what was experimentally discovered. The authors should consider condensing these sections to improve clarity and focus. *We have made major changes to the manuscript, specifically to our updated discussion that hopefully reduces the amount of redundancy.*
