# Supplementary material for: Leveraging a synthetic biology approach to enhance BCG-mediated expansion of Vγ9Vδ2 T cells
Source: PLoS One. 2026 Apr 6;21(4):e0343925. doi: 10.1371/journal.pone.0343925 (PMC13052839; doi:10.1371/journal.pone.0343925)
Supplement: Attachment — Submitted filename: Response to reviewer comments.pdf [file pone.0343925.s007.pdf]

Point-by-point response to reviewers

Thank you for your thoughtful feedback to improve our manuscript. We have addressed the lone comment of Reviewer 3 that came up during the second review regarding normalization of the input samples used for the HMPBB bioassay in Figures 2C, 3C, S1 and S2.

Reviewer #1: All concerns adequately addressed. Crazy that I have to write 100 characters in order for the website to accept this response. *Thank you!*

Reviewer #3: The revised manuscript shows clear improvement and the work is progressing in a strong direction. One specific point still requires clarification to strengthen the reproducibility of your key findings:

PBMC Expansion Assay Methodology

The PBMC stimulation workflow is appropriate; however, the description of bacterial lysate preparation would benefit from quantitative detail. At present, lysate input is reported only as dilution factors, without specifying the actual concentration of the stimulatory material. Since several processing steps (bead-beating, lysate recovery, 3 kDa filtration, volume adjustments) can alter the final composition, Bacterial culture OD normalization alone may not ensure equivalent stimulant load between samples.

Because the PBMC expansion data (e.g., Fig. 2C, 3C, S2 B–D) are central to the manuscript's conclusions regarding the differential effects of engineered rBCG versus wild-type BCG, it would substantially strengthen the study to normalize lysates to an absolute metric- such as total protein concentration (µg/mL or µg/well, measured by BCA/Bradford) or a comparable mass-based standard.

For clarity and rigor, it is recommended to include:

Quantification of lysate protein concentration prior to use (µg/mL).

Normalization of all lysates to the same protein input per well during stimulation.

This addition will enhance comparability across experimental arms and ensure that observed differences in PBMC expansion reflect biological effects rather than variation in lysate load. Addressing this point will more firmly support the manuscript's core conclusions.

*Fortunately, we saved samples of the whole cell lysate as well as the eluent from the 3kDa column used from all three biological replicates (except for the BCG+*gcpE *over-expression strain, where we only had two of the three replicates for the crude lysate). As suggested by Reviewer 3, we performed BCA assays to quantify total protein concentration in both the crude lysates and the 3kDa column eluents (which are the samples that were applied to the cells). We were able to measure proteins in these samples and there were no significant differences between WT and either engineered strain (shown below). This is consistent with our previous experience normalizing by cell numbers.* ***We have updated the methods section to include BCA normalization of lysate inputs.***

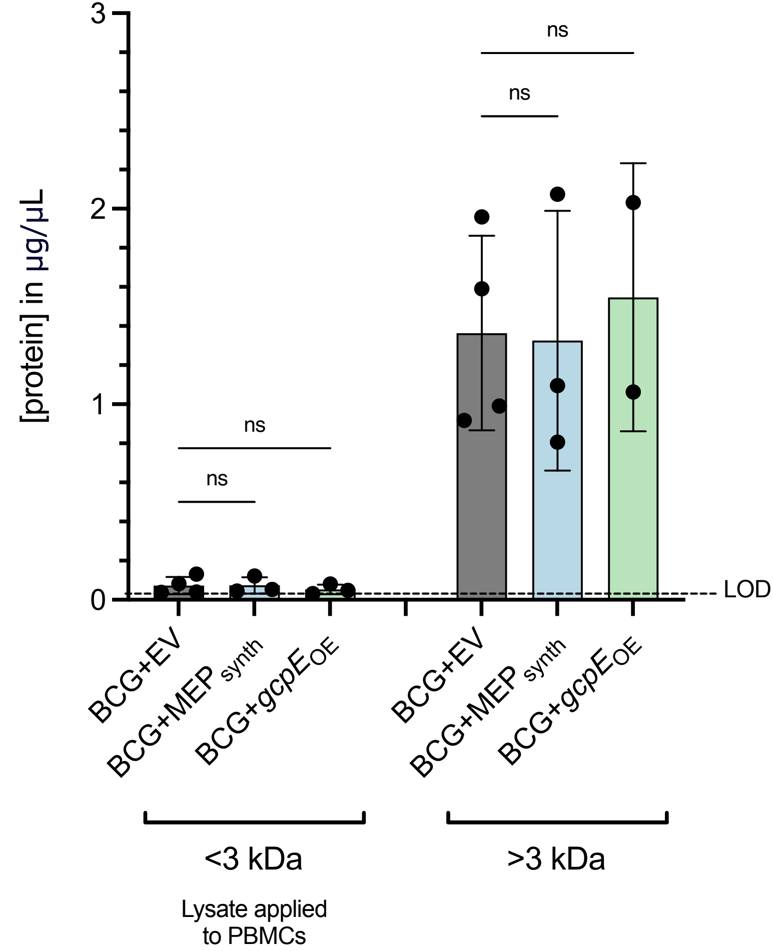

[protein] in µg/µL
3
2
1
0
ns
ns
ns
ns
LOD
BCG+EV
BCG+MEP synth
BCG+gcpE OE
BCG+EV
BCG+MEP synth
BCG+gcpE OE
<3 kDa
>3 kDa
Lysate applied to PBMCs
